# Establishment of a Modified in Vitro Cultivation of Protoscoleces to Adult *Echinococcus granulosus*; an Important Way for New Investigations on Hydatidosis

**Published:** 2012

**Authors:** T Mohammadzadeh, SM Sadjjadi, HR Rahimi, S Shams

**Affiliations:** 1 Department of Parasitology and Mycology, School of Medicine, Shiraz University of Medical Sciences, Shiraz, Iran; 2 Fars Industrial Abattoirs, Shiraz, Iran

**Keywords:** *Echinococcus granulosus*, in vitro, Reared Worm, Zoonotic, Cestode, Cultivation

## Abstract

**Background:**

*Echinococcus granulosus*, a zoonotic cestode parasite, causative agent of hydatid cyst is endemic in many parts of the world including the Middle East. Study on different aspects of this parasite is very important and valuable. However, working with adult worms which their habitat situated in the small intestine of canids, is dangerous and risky. Achieving such risky situation needs a controlled condition which is cultivation of the organisms in the laboratory. In this regard, cultivation of *E. granulosus* protoscoleces leading to adult worms was established in the laboratory for the first time in Iran.

**Methods:**

Under aseptic conditions a number of protoscoleces were cultivated in diphasic S.10E.H medium using CO2 incubator to produce adult worms.

**Results:**

Different forms of parasites including pre-segmentation stages (PS1 - PS4) and segmentation stages (S5-S8) and developing stages in segmented worms (S10-S11) were observed and evaluated in these medium. Finally adult worms contained four proglottids with a large and distinct genital pore were observed 50-55 days post cultivation. These parasites do not produce fertile eggs and conclusively do not have risk of hydatid disease transmission to the researchers.

**Conclusion:**

The mentioned method for producing *E. granulosus* adult worms can open a new window for researches and facilitate working on different aspects of hydatidosis especially for diagnosis, protection and treatment studies.

## Introduction

*Echinococcus granulosus* is an important zoonotic parasite and causative agent of hydatid cyst in human and animals. This parasite is endemic in many parts of the world including Middle East (1). The disease is responsible for almost 1% of all admissions to surgical wards in Iran (2). Regarding the importance of the disease, study on different aspects of this parasite including biology, pathogenesis, diagnosis and treatment is very important (3). However, working with *E. granulosus* which its habitat is small intestine of canids and is transmitted to human by excreted eggs from adult worms is very dangerous and risky. Achieving this problem needs application of a controlled condition in the laboratory which is cultivation of the organism. This is one of the most important methods for study on different aspects of human tissues, animal and plants organisms. It is modern and implemented in various branches of the medico-biological sciences (4).

In the present study, in vitro cultivation of protoscoleces for producing of *E. granulosus* adult worms was established for the first time in Iran with some modifications which make it easy to work in the laboratories in order to facilitate future studies regarding different aspects of this important zoonotic parasite.

## Materials and Methods

### Source of hydatid materials

A total of 50 sheep livers and lungs infected with various numbers of hydatid cysts were collected from abattoirs of Shiraz, Fars Province, Iran. The infected organs were immediately transferred to the Helminthology Research Laboratory at Shiraz University of Medical Sciences, for evaluation of cysts and further studies.

### Aseptic dissection of cysts and collection of protoscoleces (PSCs)

Collection and washing of PSCs was carried out as previously described with some modifications (5–7). Briefly, the surface of the hydatid cysts were washed using 70% ethanol and cut opened under aseptic conditions. Hydatid cyst fluid containing PSCs was collected from cysts, transferred to 50 ml Falcon tubes. Viability was checked by eosin dye (7–9) and observing flame cell movements (10). PSCs with more than 80% viability were selected, passed through two layers sterile gauze, allowed to be settled down in 50 ml Falcon tubes, washed 3 times with sterile PBS (instead Hanks's solution).

### Cultivation

Cultivation of the tubes was carried out under laminar flow hood. The culture medium was S.10E.H, consisted of two phases; liquid phase [260ml of CMRL 1066, 100 ml of fetal calf serum, 36 ml of 5% yeast extract (in CMRL 1066), 5.6 ml of 30% glucose (in distilled water), 1.4 ml of 5% dog bile (in PBS), 100U/ ml each of penicillin and streptomycin (P/S), 20 mM HEPES, 10 mM NaHCO3] and solid phase (Bovine serum coagulated at 76°C for 20-30 minutes). The basic technique was carried out as described in some details (6, 10) with some modifications. Briefly, the initial culture material consisted of washed PSCs from hydatid cyst fluid. At first PSCs were checked for sterility in liquid phase during 24-48 hrs. About 100 µl of PSCs (almost 10000 PSCs) were transferred into the culture flasks with filter containing 20 ml of the S.10E.H medium and incubated in CO2 incubator with 5% CO2 at 37°C. Additional works and modifications were as follows:

- The gas mixture (8-10% O2, 5% CO2 in N2) was also used in a number of culture flasks without filter and growth of reared helminths was compared with the worms that utilized CO2 in CO2 incubator.
- Furthermore in a short survey a number of flasks were incubated at 38.5°C instead 37°C.
- Non-commercial bovine serum, canine serum and AB+ human serum was used and compared with commercial newborn calf serum that has been used in the previous studies (6, 10).

## Results

**Fig. 1:**
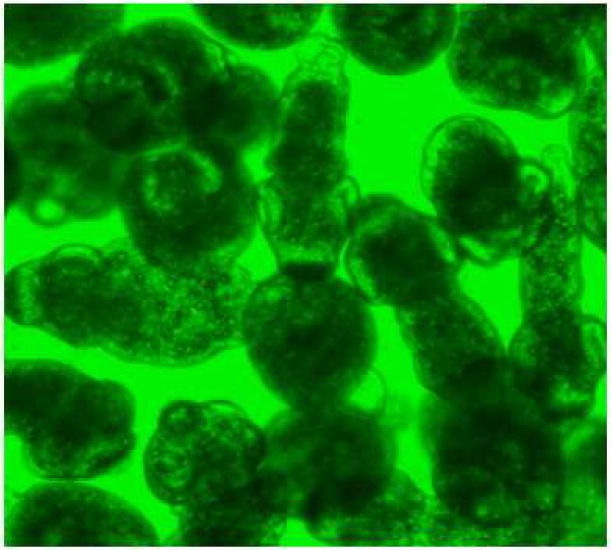
Evaginated protoscoleces (PS.1)

**Fig. 2:**
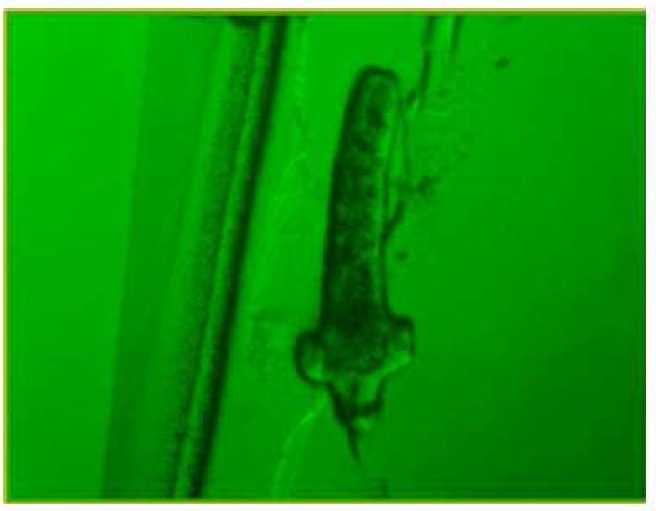
Evaginated protoscolex with excretory canal (PS.2)

**Fig. 3:**
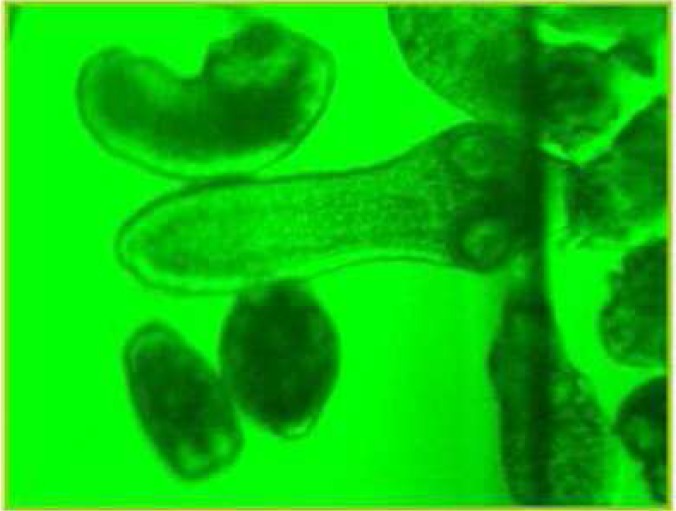
Evaginated protoscoleces with excretory canal and bladder (PS.3)

**Fig. 4:**
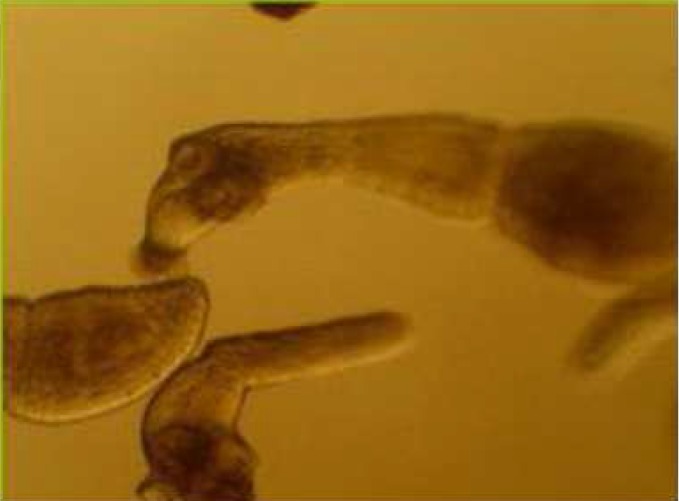
Banding form and segmentation (PS.4 & S.5)

**Fig. 5:**
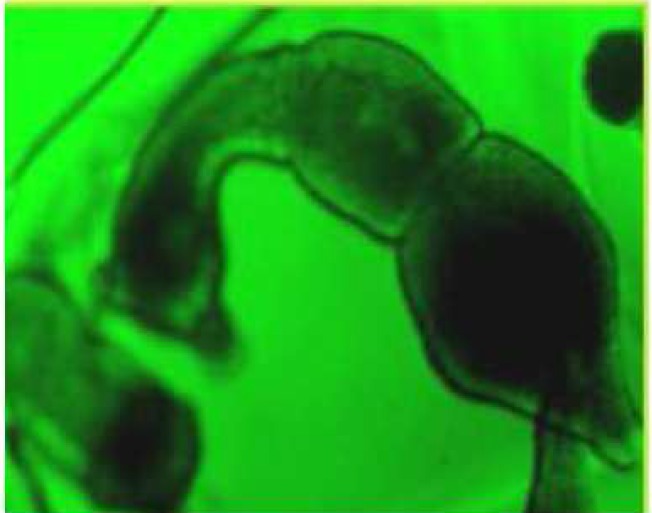
Second proglottid formed (S.6)

**Fig. 6:**
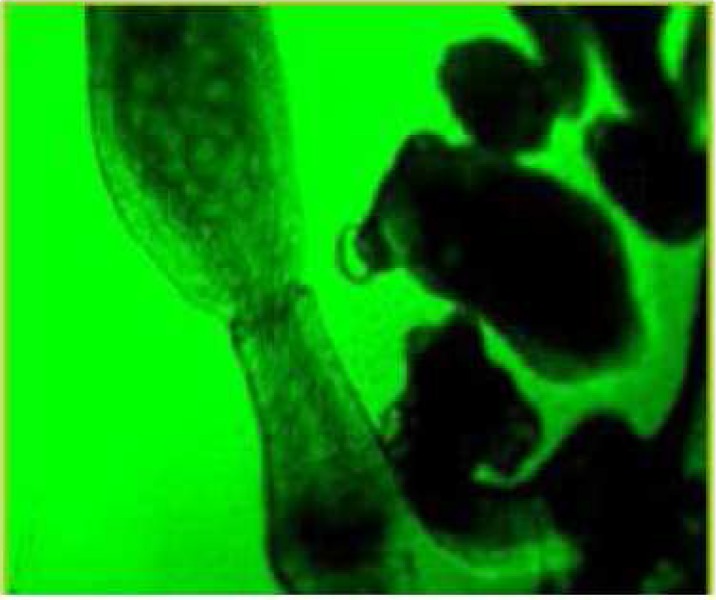
Testes appearance (S.7)

**Fig. 7:**
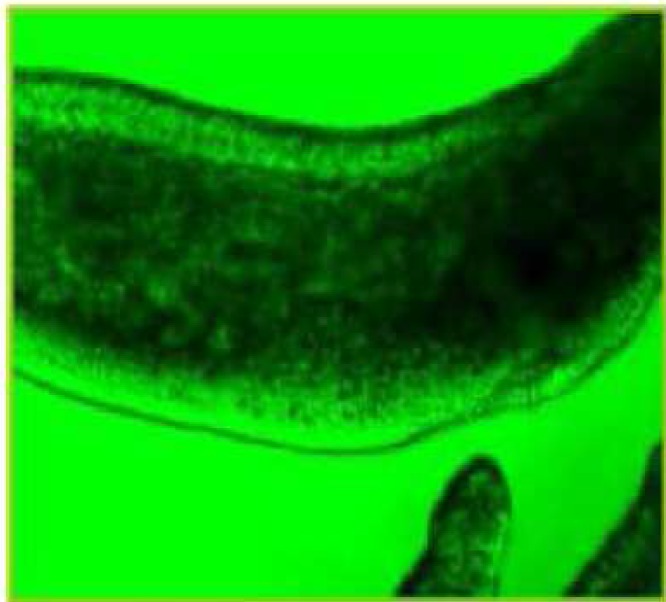
Genital pore evident (S.8)

**Fig. 8:**
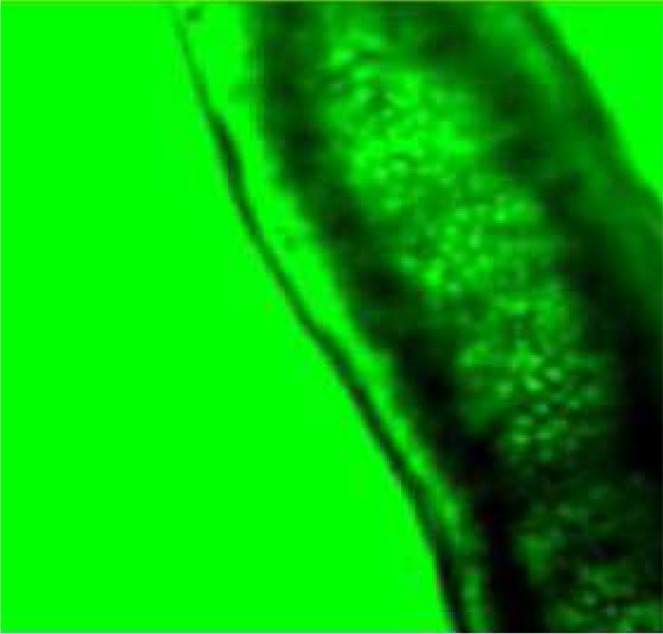
Uterus evident, Cells in uterus (S.10, S.11)

**Fig. 9:**
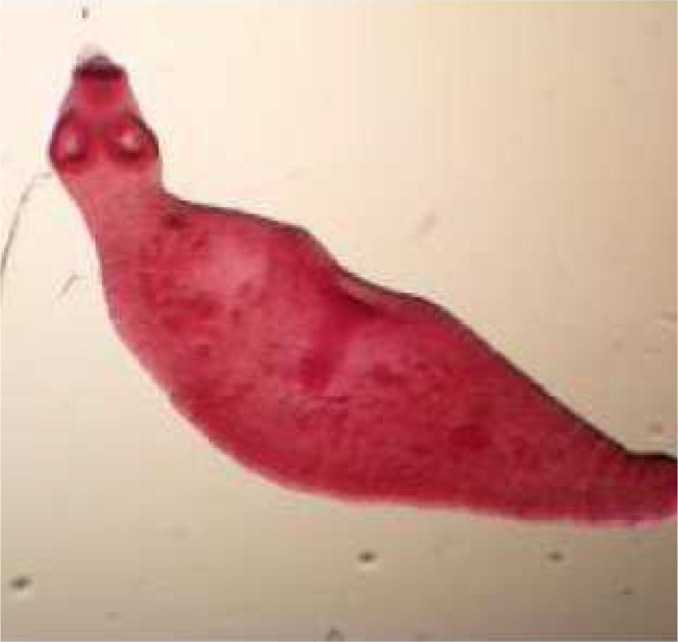
Monozoic form (stained with FAAL)

**Fig. 10:**
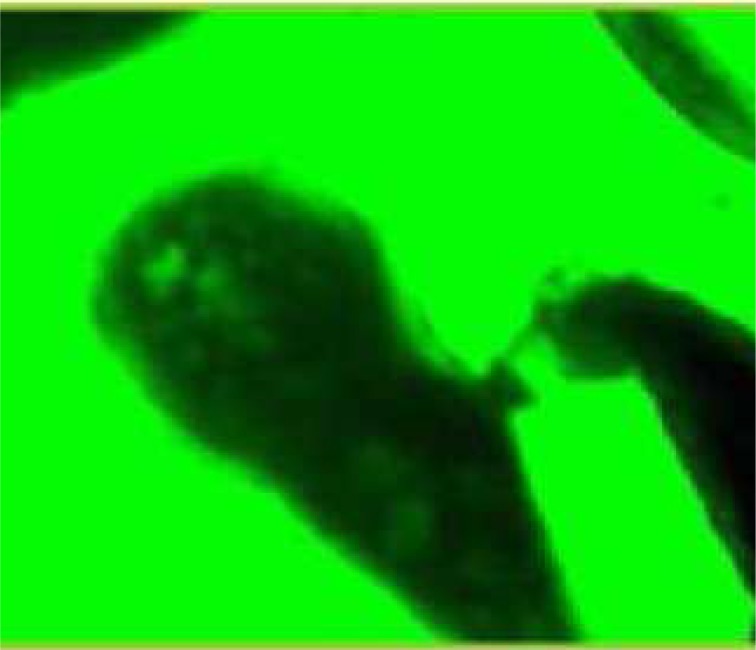
Free proglottid shed in culture medium

**Fig. 11:**
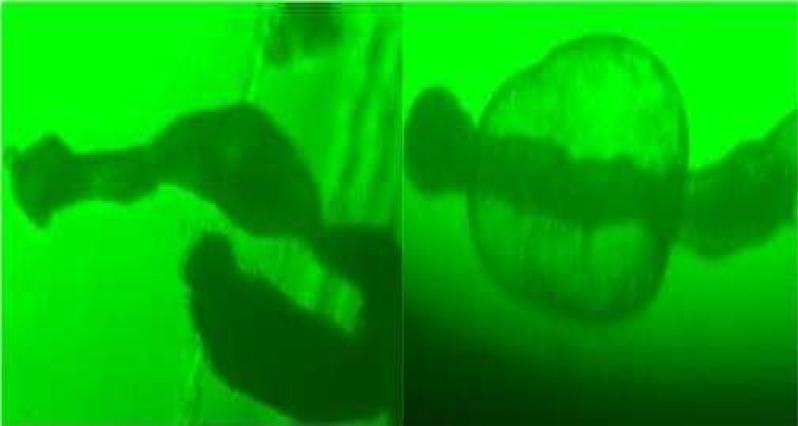
Dying parasites

**Fig. 12:**
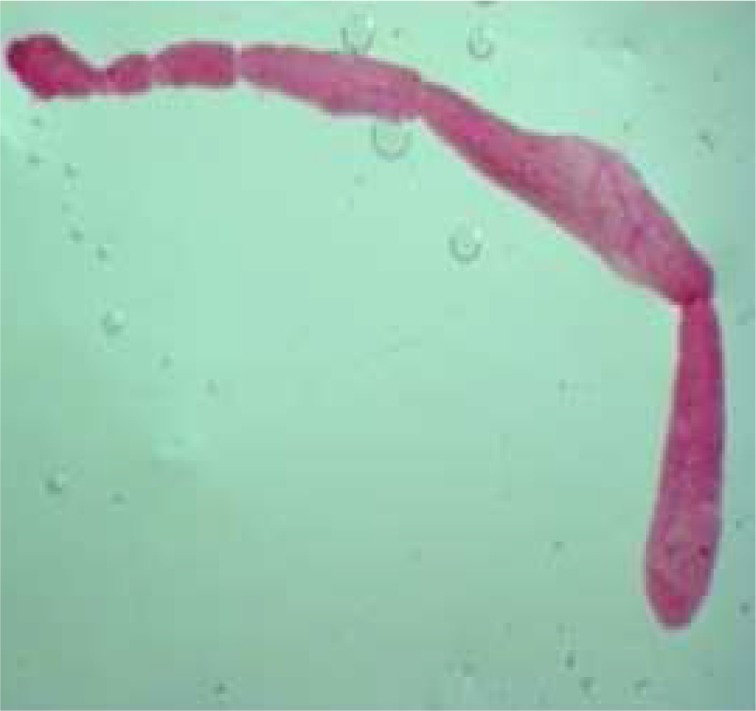
Five segmented worm (stained with FAAL)

During these assays different stages of the worms were observed from protoscoleces to adult worms as follows: evaginated prtoscoleces (24hrs after cultivation) (PS.1: Fig. 1), evaginated prtoscoleces with excretory canal (PS.2: Fig. 2), evaginated protoscoleces with excretory canal and bladder (PS.3: Fig. 3), banding form and segmentation (PS.4 and S.5: Fig. 4), second proglottid formation (S.6: Fig. 5), testes appearance (S.7: Fig. 6), genital pore evident (S.8: Fig. 7), uterus evident and cells in uterus (S.10 and S.11: Fig. 8) were observed during around 2 months. Also monozoic form (Fig. 9), free proglottid shed (Fig. 10) and dying parasites (Fig. 11) were seen in culture medium. The time of the changes from protoscoleces to adult worms are shown in Table 1. The mean size of 1, 2, 3 and 4-segmented worms was 1028±229 µm, 1974± 381µm, 2673± 410 µm and 3520±581 µm respectively. Five segmented worms (Fig. 12) were also observed in present cultivation with mean size 4710±579 µm (Table 2).

**Table 1:** Development time of *Echinococcus granulosus in vitro*

Stages of proglottids formation	Development time in day
First proglottid	17-23
Second proglottid	25-30
Third proglottid	37-40
Forth proglottid	50-55

**Table 2:** Size of *Echinococcus granulosus in vitro*

Stages of proglottids formation	Size/µm
First proglottid	1028±229
Second proglottid	1974±381
Third proglottid	2673±410
Forth proglottid	3520±581
Fifth proglottid	4710±579

During the follow-up development of the parasites a few diversity were seen. When 50%-60% of the PSCs developed completely, other parasites were in previous stages.

These condition was observed in both PSCs groups incubated at 37°C and 38.5°C. Consequently, only the incubation at 37°C was continued.

The present investigation did not show considerable differences between different types of sera that used as solid media.

There was also no valuable difference between utilizing gas mixtures (8-10% O2, 5% CO2 in N2) and CO2. However, application CO2 alone in CO2 incubator was simpler than using gas-mixture.

## Discussion

Cultivation of *E. granulosus* backs to several years ago (11). We succeeded to produce adult worms from protoscoleces in the laboratory. Selection of suitable PSCs is very important for cultivation. We examined several sheep hydatid cyst before culture for checking their viability and sterility.

Generally brood capsules contain dead tissues along PSCs. It has been suggested that brood capsules contain more than 60% viable PSCs use for cultivation (6). We selected hydatid cysts contained more than 80% viable PSCs (Free and in brood capsule).

In culture protocols, usually enzyme pretreatment is used. This protocol uses pepsin, trypsin and pancreatin for releasing the PSCs and digesting dead PSCs and tissues (5, 6, 10, 12). However, it has some problems for optimizing and can impose negative effect on viability of PSCs (6). In this regard, hydatid cyst compounds were passed through two layers sterile gauze. Although a few brood capsules may be transferred to culture media, their effect on parasite growth is not considerable. Furthermore enzyme pretreatment requires more time and is expensive in compare with physical procedure for separating the PSCs.

Pretreatment with evaginating solution containing sodium taurocholate using bile salt can induce additional difficulty, because commercially bile salts (sometimes called Sodium tauroglycocholate) contain sodium taurocholate and sodium glycocholate (6). There are high levels of sodium deoxycholate in the most of commercial bile salts. Using sodium deoxycholate can destroy tegument and kill PSCs (6, 11, 13). Therefore researchers should choose sodium taurocholate without sodium deoxycholate (6). We did not apply pretreatment solution for above reason. But PSCs were incubated in liquid phase (CMRL 1066 containing different material including bile dog) for 24-48 hrs. This stage can be used for checking sterility and adaptation PSCs with bile dog for evagination.

Although our media contains bile dog instead sodium taurocholate and despite bile dog may be have some variation from dog to dog (6), we could not find considerably problems in our cultures.

A mixture of gas (8-10% O2, 5% CO2 in N2) has also been used for culture media (5, 6, 10, 12). However critical evidence about using it has not been reported (10). In the present study application gas-mixture and CO2 was evaluated separately. Our experience showed that optimizing of dose of gas mixture is somewhat difficult. Un- suitable dose of gas could also impose negative effect on culture. So we continued work with 5% CO2.

A number of PSCs were also evaluated at 38.5°C instead 37°C for a short time. During this time we could not find considerable differences between them.

Smyth et al. used penicillin and streptomycin in culture media. In addition, they utilized tetracycline and mycostatin for resistant strains and preventing of fungi growth respectively (5). Gentamicin has also been applied (14). A mixture of P/S and /or gentamicin has been used too (10). In our study 150µg/ml of each P/S was used. Sometimes 100 µg/ml gentamicin has been added to 100 µg/ml of P/S.

It has been reported that calf serum is as good as or even better than bovine serum, although critical assessment has not been possible (6). Utilizing different batches of serum can have different effect on worm development (14). In the present study, we have used both commercial newborn calf serum and non-commercial bovine serum. For dissolving this issue usually pooled bovine serum was used. In general we did not find any problem using separately or both sera.

Furthermore we compared different sera samples including AB+ human sera which are a new experience as well as canine and bovine serum as the solid phase. There were no valuable differences between all of them. Therefore we speculated that any serum which is more accessible can be used for the culture.

Physical condition of the cyst based on transport condition, strain of parasite and genetic factors (5) are effective on evagination. In addition some unexplained relationship between rate of the parasites and the volume and shape of culture vessel and the culture medium can effect on growth of parasites, although it is difficult to be proved (6).

Our experiences show that genetic factors and number of cultivated parasites may play the most important role in parasite development. In addition some unexplained or unexpected factors such as culture vessel may have some roles.

The follow-up development of the parasites showed some diversity during cultivation time such that when 50%-60% of PSCs developed completely, other parasites were in previous stages.

Optimum development of *E. chinococcus granulosus* in dog includes: segmentation 14 day, 2nd proglottid formation 17 days, testes appearance 22 days, uterus appearance 28 days, cells in uterus 32 days and fertile eggs in uterus 40 days (6). It is different in parts by the reared worms in the culture. So that, Smyth and Davis have found that out of 35 cultures only 1 case had first segment within 15 days, about 50% of experiments were showed segmentation within 16-22 days and rest of them had this phenomenon during more than 22 days (6).

In our study PSCs were selected with more than 80% viability. A total of 70%-80% of them were evaginated within 1-3 days. First segmentation observed during 17-23 days post cultivation. Second and third segmentations were observed 25-30 and 37-40 days post cultivation respectively. Fourth proglottid with a large, distinct genital pore was also seen during 50-55 days post cultivation (Table 1). Genital pore was opened and closed rhythmically in this segment.

Despite major advances in cultivation of *E. granulosus* adult worm from PSCs to mature strobilate tape worms, fertilization has not yet been reported (6, 10). In other studies thick shelled eggs was produced from partially developed adult worms separated from dog intestine, but only oncospheral motility has evaluated (15, 16). Kumaratilake and Thompson have evaluated infectivity of these eggs by infecting mice (17). Recently sexual maturation of *E. granulosus* adult worm was established in alternative definitive host, Mongolian gerbil (*Meriones unguiculatus*) (18). In the present study uterus containing free cells was found but fertile eggs have never been seen in cultivated media. This phenomenon could provide a suitable and safe condition for different branches of investigation regarding studies with adult form of this important parasite.

The size of adult worms usually is less than 6mm (19). The maximum size of a 3- segmented cultivated worm has been reported 2000µm and it is somewhat similar to 2- segmented worm grew in a dog (4). Maximum size of three and four segmented worms was 2.94 and 3.2 mm respectively (20). Length of mature parasites that maintained in gerbil was 3.2 mm (18).

Maximum size of three and four segmented worms in our study was 3.5 and 4.7 mm respectively. Five segmented worms were also observed in our study which seems to be interesting result for *in vitro* cultivation of protoscoleces to adult forms of *Echinococcus granulosus*. Maximum size of these worms measured to be 5.12 mm. A number of researchers believe that different size in different studies may reflect strain difference (20).

In conclusion, *E. granulosus* adult worms were established in our laboratory with a simple and relatively cheap situation comparing to the original procedures. Regarding the result of this study, the reared parasites do not produce fertile or mature eggs. Hensley, they have not any risk of infection for researchers. The mentioned method achieving adult *E. granulosus* in culture media can open a new window for researchers to work on different aspects of hydatidosis especially diagnosis, protection (21) and treatment of this important parasitic zoonoses.
